# Membranoproliferative glomerulonephritis: current histopathological classification, clinical profile, and kidney outcomes

**DOI:** 10.1590/2175-8239-JBN-2022-0016en

**Published:** 2022-06-27

**Authors:** Thaíza Passaglia Bernardes, Gianna Mastroianni-Kirsztajn

**Affiliations:** 1 Universidade Federal de São Paulo, Divisão de Nefrologia, São Paulo, SP, Brasil.

**Keywords:** Pathology, Glomerulonephritis, Glomerulonephritis, Membranoproliferative, Lupus erythematosus, systemic, Hepatitis C, Patologia, Glomerulonefrite, Glomerulonefrite Membranoproliferativa, Lúpus Eritematoso Sistêmico, Hepatite C

## Abstract

**Introduction:**

Membranoproliferative glomerulonephritis (MPGN) is a rare glomerular disease with a variable prognosis. A new classification based on the presence or absence of immunoglobulins and complement deposits in immunofluorescence microscopy (IF) of kidney biopsy has recently been proposed. The objectives of the study were to determine and compare the clinical, laboratory, and histopathological characteristics of those with primary or secondary MPGN, reclassify the primary ones based on IF findings, and evaluate kidney outcomes.

**Methods:**

This was an observational retrospective cohort study carried out in a single center (UNIFESP), based on the data collected from medical records of patients followed from 1996 to 2019.

**Results:**

Of 53 cases of MPGN, 36 (67.9%) were classified as primary and 17 (32.1%) as secondary MPGN. Most patients were hypertensive (84.9%) and had edema (88.7%) and anemia (84.9%); 33 (91.7%) patients classified as primary MPGN were reclassified as immune-complex-mediated and 3 (8.3%) as complement-mediated. The secondary MPGN group had hematuria more frequently (p <0.001) and a higher prevalence of deposits of IgG (p = 0.02) and C1q (p = 0.003). Regarding the outcome, 39% of the patients achieved partial or complete remission. Lower initial serum albumin and higher initial 24-hour proteinuria were factors associated with worst renal prognosis.

**Conclusions:**

According to the new histological classification, the vast majority of MPGN cases were classified as being mediated by immune complexes. There were few differences between primary and secondary MPGN in relation to their clinical and laboratory characteristics.

## Resumo

**Introdução:**

Glomerulonefrite membranoproliferativa (GNMP) é uma doença glomerular rara com prognóstico variável. Recentemente, foi proposta uma nova classificação baseada na presença ou ausência de imunoglobulinas e depósitos de complemento na microscopia de imunofluorescência (IF) da biópsia renal. Os objetivos do estudo foram determinar e comparar as características clínicas, laboratoriais e histopatológicas daqueles com GNMP primária ou secundária, reclassificar as primárias com base em achados da IF e avaliar os desfechos renais.

**Métodos:**

Este foi um estudo de coorte observacional retrospectivo realizado em centro único (UNIFESP), com base nos dados coletados de prontuários de pacientes acompanhados de 1996 a 2019.

**Resultados:**

Dos 53 casos de GNMP, 36 (67,9%) foram classificados como GNMP primária e 17 (32,1%) como GNMP secundária. A maioria dos pacientes era hipertensa (84,9%) e apresentava edema (88,7%) e anemia (84,9%); 33 (91,7%) pacientes classificados como GNMP primária foram reclassificados como mediados por imunocomplexo e 3 (8,3%) como mediados por complemento. O grupo de GNMP secundária apresentou mais frequentemente hematúria (p <0,001) e maior prevalência de depósitos de IgG (p = 0,02) e C1q (p = 0,003). Com relação ao desfecho, 39% dos pacientes alcançaram remissão parcial ou completa. Albumina sérica inicial mais baixa e proteinúria de 24 horas inicial mais elevada foram fatores associados a pior prognóstico renal.

**Conclusões:**

De acordo com a nova classificação histológica, a grande maioria dos casos de GNMP foram classificados como sendo mediados por imunocomplexos. Houve poucas diferenças entre GNMP primária e secundária em relação às suas características clínicas e laboratoriais.

## Introduction

Membranoproliferative glomerulonephritis (MPGN) accounts for approximately 7-10% of all cases of biopsied glomerulonephritis^1,2^. In Brazil, according to previous studies, this prevalence was 3.4-9%. The largest study was conducted in São Paulo with 9617 kidney biopsies from all over the country, with a prevalence of 5.2% of MPGN among primary glomerulonephritis^3^. Some studies in the last decades have shown that the frequency of MPGN is decreasing, especially in developed countries, and suggested that this decline is related to a better control of infectious diseases^4,5^.

Recently, a new classification based on the underlying pathogenetic process was proposed according to immunofluorescence microscopy (IF) findings. MPGN was thus classified as being mediated by immune complexes, complement dysregulation, or mechanisms not involving immunoglobulin or complement deposition^1,2^. The condition was divided into three groups: presence of immunoglobulins and C3, presence of C3 without immunoglobulins, and absence of C3 and immunoglobulins^6^.

Immune-complex-mediated MPGN occurs in chronic infections, autoimmune diseases, monoclonal gammopathies, or idiopathic forms. An underlying cause can be found most of the time^7-9^. Among the associated infections, the most frequent are hepatitis B and hepatitis C, and among autoimmune diseases, the most prevalent is systemic lupus erythematosus (SLE)^2, 7-9^.

The clinical presentation of MPGN is quite variable, ranging from a slow and progressive course to rapidly progressive glomerulonephritis. As it is a rare glomerulonephritis, there are relatively few studies on outcomes and therapy, contributing to difficult clinical management. Therefore, better characterization of course and prognosis can provide a rationale for clinical investigation and disease-specific treatments.

## Methods

An observational retrospective cohort study was carried out in a single Brazilian center that is reference for Nephrology. The study was based on data from the medical records of patients followed at UNIFESP-São Paulo from 1996 to 2019 who were diagnosed with MPGN (by light microscopy and immunofluorescence techniques), and classified as primary or secondary MPGN.

The inclusion criterion was diagnosis of MPGN (primary or secondary) in native kidneys in patients with at least six months of follow-up in our service. The exclusion criteria were absence of renal biopsy report in the medical record, absence of IF analysis, or insufficient data for the purpose of this study.

In both primary and secondary MPGN, the parameters evaluated included clinical and epidemiological aspects and laboratory data at the time of diagnosis (shown in Table 1) as well as serology tests for hepatitis B, hepatitis C, HIV, and syphilis. In addition, at the end of the follow-up, the levels of proteinuria (g/24h), serum albumin (g/dL) and creatinine (mg/dL), hemoglobin (g/dL), and estimated GFR were again analyzed. Estimated GFR was calculated using CKD-EPI for patients older than 16 years and Schwartz equations for patients younger than 16 years.

**Table 1:** Demographic, clinical and laboratory characteristics of the patients according to the group of membranoproliferative glomerulonephritis

	Total (N=53)	Primary (N=36)	Secondary (N=17)	P
Male gender, n (%)	26 (49.1)	15 (41.7)	11 (64.7)	0.117^a^
Age (years)	53 ± 15.1	38.1 (±16.3)	40.4 (±12.4)	0.675^b^
Hypertension, n (%)	45 (84.9)	30 (83.3)	15 (88.2)	>0.999^c^
Edema, n (%)	47 (88.7)	31 (86.1)	16 (94.1)	0.651^c^
Anemia, n (%)	45 (84.9)	29 (80.6)	16 (94.1)	0.412^c^
Nephrotic syndrome, n (%)	15 (33)	10 (32.3)	5 (35.7)	>0.999^a^
Initial SAlb (g/dL)	2.8 ± 0.7	2.8(±0.7)	2.8 (±0.7)	0.740^d^
Hematuria, n (%)	50 (94.3)	33 (91.7)	17 (100)	0.525^c^
Initial 24hP (g/24h)	5.2 (±4.6)	5.5 (±5.2)	4.5 (±2.9)	0.935^b^
Initial SCr (mg/dL)	1.5 (±1.0)	1.4 (±0.9)	1.7 (±1.1)	0.360^b^
eGFR (mL/min/1.73m^2^)	66.6 (±36.3)	69.2 (±36.9)	61.2 (±35.4)	0.293^b^
Low serum C3 level	24 (49.0)	16 (48.5)*	8 (50)**	0.921^a^
Low serum C4 level	23 (46.9)	13 (39.4)*	10 (62.5)**	0.129^a^
Final SAlb (g/dL)	3.7(±0.6)	3.6(±0.6)	3.8 (±0.6)	0.218^d^
Final 24hP (g/24h)	3.2(±3.8)	3.6 (±3.8)	2.4 (±3.7)	0.257^b^
Final SCr (mg/dL)	2.4 (±2.3)	2.6 (±2.4)	2.0 (±1.9)	0.435^b^
eGFR < 15mL/min	7 (13.2)	5 (13.9%)	2 (11.8%)	>0.999^c^
SCr 2x initial level	15 (28.3)	13 (36.1%)	2 (11.8%)	0.103^c^
Follow up time	6.3 (±7.4)	6.3 (±5.5)	6.2 (±10.6)	0.182^b^

Values for continuous variables are expressed as mean ± SD. aPearson's Chi-Square, bMann-Whitney, cFisher's exact, dStudent’s t-test for independent samples. SAlb: serum albumin; SCr: serum creatinine; 24hP: 24-hour proteinuria; eGFR: estimated glomerular filtration rate (CKD-EPI).

* 33 patients analyzed;

** 16 patients analyzed.

The IF findings of renal biopsy of patients with primary MPGN were also analyzed to reclassify the biopsies according to the new classification criteria.

A descriptive statistical analysis was initially performed. The comparison of proteinuria in different treatment regimens between primary and secondary MPGN was done using the Student’s t-test for independent samples, Mann-Whitney, Chi Square test and Fisher’s exact test, and Kruskall-Wallis and Fisher’s exact tests. An alpha of 5% was used to indicate significance.

## Results

**Figure 1:**
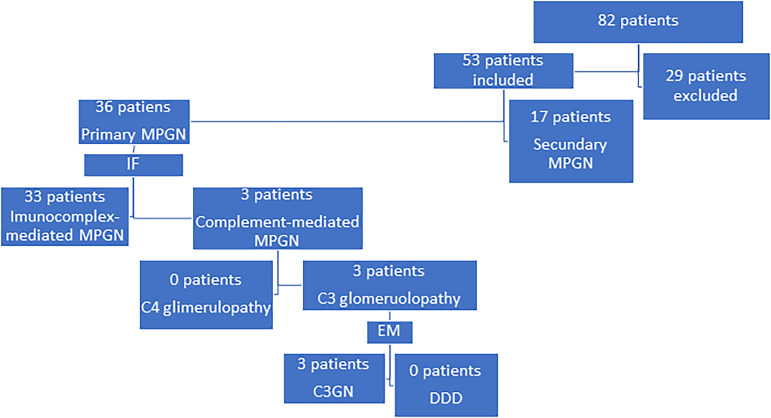
Classification of the study patients.

IF: immunofluorescence microscopy; EM: electronic microscopy; C3GN, C3 glomerulonephritis; DDD, dense deposit disease.

Eighty-two cases diagnosed with MPGN were identified in the medical records, and after applying the exclusion criteria, the sample selected in this research was of 53 cases of MPGN, of which 36 (67.9%) were classified as primary and 17 (32.1%) as secondary (Figure 1) The mean age of patients at the beginning of the follow-up was 38.8 years. Most patients were hypertensive (84.9%), defined as a systolic blood pressure of 140 mmHg or more, or a diastolic blood pressure of 90 mm Hg or more, had edema (88.7%), and anemia (84.9%), defined as hemoglobin < 13g/dL for men or <12g/dL for women; 33 (91.7%) primary MPGN were reclassified as with immune-complex-associated MPGN and 3 (8.3%) as associated with complement deposition.

The secondary MPGN group had higher rates of hematuria, defined as the presence of >10.000 erythrocytes/mL of urine (p <0.001) and a higher prevalence of IgG (p = 0.02) and C1q (p = 0.003) deposits compared to the primary group. Regarding the outcome, final serum albumin <3g/dL and final 24-hour proteinuria >3.5g/24h were associated with worse kidney prognosis.

The most common cause of secondary MPGN was SLE, which accounted for 47% of the cases, followed by hepatitis C virus infection (29.4%) and schistosomiasis (17.6%).

As summarized in Table 1, approximately half of the patients were male (49.1%) and the average age of patients at the beginning of the follow-up was 38.8 years, ranging from 13.2 to 61.9 years. Most patients were hypertensive (84.9%), had edema (88.7%), anemia (84.9%), and hematuria (94.3%). Other features of the diagnosis are shown Table 1. Mean proteinuria was 5.2 ± 4.6 g/day, mean serum albumin was 2.8 ± 0.7 g/dL, mean eGFR was 66.6 mL/min/1.73 m^2^, and mean serum creatinine was 1.5 mg/dL. Low serum C3 levels were present in 49.0%, and low C4 in 46.9% of the patients.

All kidney biopsy were evaluated by IF microscopy, and deposits of C3 were found in 100% of the biopsies. A high prevalence of deposits of IgM (75%), IgG (66%), lambda (55.3%), kappa (55.3%), and C1q (50%) was also observed. IgA was the least prevalent deposit, being positive in 28.3% of biopsies. All patients in the group with primary MPGN tested negative for HCV, HBsAg, HIV, and VDRL tests and 4 had isolated positive ANA. Based on the new classification, 33 (91.7%) of the primary MPGN were classified as immune-complex-associated MPGN and 3 (8.3%) as associated only with complement. After electron microscopy analysis, the last 3 were diagnosed as C3 glomerulonephritis.

The group of secondary MPGN had a higher frequency of hematuria (p <0.001) and IgG (p=0.02) and C1q deposits (p=0.003). As shown in Table 1, other characteristics did not differ between both groups.

Of the 51 patients from whom it was possible to assess the response to treatment, 10 had complete remission (final 24-hour proteinuria <0.3 g/24h, without increasing serum creatinine levels), 10 had partial remission (at least 50% decrease in final 24-hour proteinuria compared with the initial values without increasing serum creatinine levels), and 31 (60.8%) had no response. In summary, there was an association between response to treatment and higher initial serum albumin (p=0.03), lower initial 24-hour proteinuria (p=0.03), and IgM deposits in the IF (p=0.02). It is also important to highlight that the group that did not show remission (complete or partial) more frequently had the kidney outcome “doubling the initial serum creatinine (Cr 2x initial value)” (p = 0.001) and progression to end-stage renal disease (ESRD) (p=0.004).

## Discussion

Glomerulonephritis with a membranoproliferative histological pattern are rare conditions and their frequency has been decreasing over the years, especially in developed countries, and this decline might be related to a better control of infectious diseases^4,5^. In our center, over the period analyzed, 82 patients were diagnosed with GNMP and 53 were evaluated in this study.

Primary MPGN was routinely characterized by medical history and negative serological tests for syphilis, HCV, HBsAg, and HIV, as well as negative ANA or isolated positive ANA without any clinical manifestation of systemic disease. It is of note that few patients were evaluated by serum protein electrophoresis and urinary protein immunofixation tests for diagnosis of gammopathies. These last tests were not routinely available in our center, being requested only in case of clinical suspicion of paraproteinemia. It is possible that the unavailability of some tests has contributed to the higher number of MPGN without an identified cause.

The most common cause of secondary MPGN was SLE, corresponding to 47% of cases, followed by hepatitis C virus (29.4%) and schistosomiasis (17.6%). In another study by Lopes *et al*.^5^, also at UNIFESP, with 17 patients with secondary MPGN, but excluding patients with SLE, the main cause was schistosomiasis, found in 64.7% of cases. It is worth noting that this study was carried out in the 1990s and the lower prevalence of hepatitis C could be justified by the fact that not all patients had been tested for such disease. When we analyzed kidney biopsies based on the new classification, IF revealed that the vast majority (94.3%) of patients with MPGN had deposition of immune complexes, while only 5.6% had exclusive C3 deposits, and there were no cases without any deposition of immunoglobulins or complement. Despite the lack of large studies of patients with MPGN and the new classification, our sample presented characteristics similar to the study by Woo *et al*.^10^, showing predominance of immune-complex-mediated MPGN. In such study, complement-mediated MPGN was found in 4.3% and immune-complex-mediated MPGN in 95.7% of the 44 patients studied.

Due to the small number of cases diagnosed as MPGN with exclusively complement deposits, it was not possible to make comparative analyzes between groups based on their IF findings.

Levin *et al*.^11^ described MPGN affecting mainly children and young adults. Our center assists patients 12 years old or older, explaining at least in part why our sample had a higher average age (38.8 years), varying from 16.7 to 66.2 years. Brigani *et al*.^12^ also observed in a study carried out in Australia in 2001 a predominance of the disease in adults, with a peak incidence in individuals aged 55-74 years.

In another study carried out by Lopes *et al*.^5^ with 41 patients with type I MPGN, a high prevalence rate of hypertension was observed (88%), as in our study, differing from previous studies that described a 33% to 38% prevalence of hypertension^13,14^. This finding could be explained by the supposedly more advanced disease stage at diagnosis of our patients, due to, for example, the difficulty of accessing the health system in our country, compared to other studies that were carried out in Europe.

There are limited data available on MPGN in Brazilian patients. Comparing with another study carried out with 92 MPGN cases in Brazil^15^, there were similar as well as conflicting data. In our study, 49% were male and the mean age was 38.8 years and in the previous study, 62% were male and the mean age was 44.3 years. Mean serum creatinine was also similar; while ours was slightly better, 1.57 mg/dL (estimated GFR of 66.6 mL/min/1.73m2), theirs was 1.8 mg/dL (estimated GFR of 41.5 mL/min/1.73m2). Both had elevated proteinuria (our average proteinuria was 5.26 g/24h with a serum albumin of 2.84 g/dL and theirs was 6.2 g/24h with a serum albumin of 2.5 g/dL). Our study population had a higher frequency of hypertension (84.9% vs. 55.4%) and hematuria at diagnosis (94.3% vs. 71.7%).

An important goal of the present study was to compare the clinical and laboratory characteristics between the groups with primary MPGN and secondary MPGN. However, few differences were found, calling attention to the more intense and frequent hematuria in the group with secondary MPGN, the same happening in relation to deposits of IgG and C1q in IF analysis. Thus, our results corroborate the idea advocated by some that the classification through IF may be the only one capable of differentiating primary and secondary MPGN^16^.

When evaluating the outcomes, we observed that most patients (60.8%) did not meet the criteria of partial or complete remission. Despite this, the majority also did not show a significant worsening of serum creatinine in the course of the disease (71.7% did not double serum creatinine levels) and only 13.2% evolved with GFR <15 mL/min/1.73m^2^. This can be justified by the fact that the majority of these patients had a GFR >60 mL/min/1.73m^2^ at diagnosis, which could be associated with a better prognosis. Patients had different follow-up times with a mean of 6.3 years.

In our study, worse prognosis was associated with the presence of nephrotic syndrome (defined as the presence of 24-hour proteinuria equal to or > 3.5 g/24h with albumin < 3 g/dL and edema), elevated 24-hour proteinuria (defined as the presence of 24-hour proteinuria equal to or > 0.3 g/24h) and low initial serum albumin. In previous studies, factors associated to a poor prognosis included nephrotic syndrome, elevated serum creatinine, and hypertension. On the other hand, patients with non-nephrotic proteinuria and normal blood pressure seem to have a better kidney prognosis in the long term^17^. We did not find any association with hypertension, possibly due to the high prevalence of hypertension in our sample. In addition, hematuria levels were not associated with worse kidney prognosis. In fact, it has been described that higher degrees of hematuria suggest more inflammation, but there is no evidence of an independent effect on prognosis^18, 19^.

Previous reports have suggested a relatively poor kidney prognosis in patients with apparently idiopathic MPGN^17-20^. However, these reports should be interpreted with caution, as they reflect a different time when some diagnostic resources were not available. In our study, there was no difference in kidney prognosis between primary and secondary MPGN.

Finally, with regard to clinical and laboratory data, the MPGN groups were distinguished only by higher hematuria in the secondary group, which in clinical practice can help in the diagnostic suspicion but does not allow differential diagnosis. Considering the classification based on IF, there was a higher frequency of IgG and C1q deposits in the secondary MPGN. Our findings support that the IF classification contributes to distinguish primary and secondary MPGN.

## Conclusion

According to the new classification based on IF findings, the vast majority of MPGN cases were classified as immune complex-mediated MPGN. There were few differences between primary and secondary MPGN regarding prognosis and their clinical and laboratory characteristics, particularly greater hematuria and higher prevalence of IgG and C1q deposits in the secondary group, corroborating the benefits of a classification based on IF findings to distinguish the two groups.

## References

[1] Masani N, Jhaveri KD, Fishbane S. (2014). Update on membranoproliferative GN. Clin J Am Soc Nephrol.

[2] Sethi S, Fervenza FC. (2012). Membranoproliferative glomerulonephritis - a new look at an old entity. N Engl J Med.

[3] Polito MG, Moura LA, Kirsztajn GM. (2010). An overview on frequency of renal biopsy diagnosis in Brasil: clinical and pathological patterns based on 9617 native kidney biopsies. Nephrol Dial Transplant.

[4] Kirsztajn GM, Vieira OM, Abreu PF, Sens W, Sens YAS. (2005). Tratamento das glomerulopatias primárias. J Bras Nefrol.

[5] Lopes LMV. Evolução da função renal em pacientes com glomerolonefrite membranoproliferativa tipo I.

[6] Cook HT, Pickering MC. (2014). Histopathology of MPGN and C3 glomerulopahies. Nat Rev Nephrol.

[7] Zand L, Fervenza FC, Nasr SH, Sethi S. (2014). Membranoproliferative glomerulonephritis associated with autoimmune diseases. J Nephrol.

[8] Sethi S, Zand L, Leung N, Smith RJ, Jevremonic D, Herrmann SS (2010). Membranoproliferative glomerulonephritis secondary to monoclonal gammopathy. Clin J Am Soc Nephrol.

[9] Yamabe H, Johnson RJ, Gretch DR, Fukushi K, Osawa H, Miyata M (1995). Hepatitis C virus infection and membranoproliferative glomerulonephritis in Japan. J Am Soc Nephrol.

[10] Woo SA, Ju HY, Kwon SH, Lee JH, Choi SJ, Han DC (2014). Reanalysis of membranoproliferative glomerulonephritis patients according to the new classification: a multicenter study. Kidney Res Clin Pract.

[11] Levin A. (1999). Management of membranoproliferative glomerulonephritis: evidence-based recommendations. Kidney Int Suppl.

[12] Briganti EM, Dowling J, Finlay M, Hill PA, Jones CL, Kincaid-Smith PS (2001). The incidence of biopsy-proven glomerulonephritis in Australia. Nephrol Dial Transplant.

[13] Belgiojoso GB, Tarantina A, Colasanti G, Bazzi C, Guerra L, Durante A. (1977). The prognostic value of some clinical and histological parameters in membranoproliferative glomerulonephritis (MPGN): report of 112 cases. Nephron.

[14] Magil AB, Price JDE, Bower G, Rance CP, Huber J, Chase WH. (1979). Membranoproliferative glomerulonephritis type I: comparison of natural history in children and adults. Clin Nephrol.

[15] Dias CB, Testagrossa L, Jorge L, Malheiros D, Woronik V. (2017). Características clínicas e histológicas de pacientes com glomerulonefrite membranoproliferativa classificadas por achados de imunofluorescência. J Bras Nefrol.

[16] Fervenza FC, Sethi S, Glassock RJ. (2012). Idiopathic membranoproliferative glomerulonephritis: does it exist?. Nephrol Dial Transplant.

[17] Somers M, Kertesz S, Rosen S, Herrin J, Colvin R, Carreta NP (1995). Non-nephrotic children with membranoproliferative glomerulonephritis: are steroids indicated?. Pediatr Nephrol.

[18] Cameron JS, Turner DR, Heaton J, Williams DG, Ogg CS, Chantler C (1983). Idiopathic mesangiocapillary glomerulonephritis. Comparison of types I and II in children and adults and long-term prognosis. Am J Med.

[19] D’Amico G, Ferrario F. (1992). Mesangiocapillary glomerulonephritis. J Am Soc Nephrol.

[20] Donadio JV, Offord KP. (1989). Reassessment of treatment results in membranoproliferative glomerulonephritis, with emphasis on life-table analysis. Am J Kidney Dis.

